# Spatially resolved CO_2_ carbon stable isotope analyses at the microscale using Raman spectroscopy

**DOI:** 10.1038/s41598-023-44903-z

**Published:** 2023-10-29

**Authors:** Samantha Remigi, Maria-Luce Frezzotti, Andrea Luca Rizzo, Rosario Esposito, Robert J. Bodnar, Andres Sandoval-Velasquez, Alessandro Aiuppa

**Affiliations:** 1 Dipartimento di Scienze dell’Ambiente e della Terra, Università Milano-Bicocca, Piazza della Scienza 4, 20126 Milan, Italy; 2 Department of Geosciences, Virginia Tech, 926 West Campus Drive, Blacksburg, VA 24061 USA; 3 Dipartimento di Scienze della Terra e del Mare, Università di Palermo, Via Archirafi 36, 90123 Palermo, Italy

**Keywords:** Geochemistry, Petrology

## Abstract

Measuring the carbon stable isotope ratio (^13^C/^12^C, expressed as δ^13^C_CO2_) in geogenic CO_2_ fluids is a crucial geochemical tool for studying Earth's degassing. Carbon stable isotope analysis is traditionally performed by bulk mass spectrometry. Although Raman spectroscopy distinguishes ^12^CO_2_ and ^13^CO_2_ isotopologue bands in spectra, using this technique to determine CO_2_ isotopic signature has been challenging. Here, we report on in-situ non-destructive analyses of the C stable isotopic composition of CO_2_, applying a novel high-resolution Raman configuration on 42 high-density CO_2_ fluid inclusions in mantle rocks from the Lake Tana region (Ethiopia) and El Hierro (Canary Islands). We collected two sets of three spectra with different acquisition times at high spectral resolution in each fluid inclusion. Among the 84 sets of spectra, 58 were characterised by integrated ^13^CO_2_/^12^CO_2_ band area ratios with reproducibility better than 4‰. Our results demonstrate the determination of δ^13^C_CO2_ by Raman spectroscopy in individual fluid inclusions with an error better than 2.5 ‰, which satisfactorily matches bulk mass spectrometry analyses in the same rock samples, supporting the accuracy of the measurements. We thus show that Raman Spectroscopy can provide a fundamental methodology for non-destructive, site-specific, and spatially resolved carbon isotope labelling at the microscale.

## Introduction

**Figure 1:**
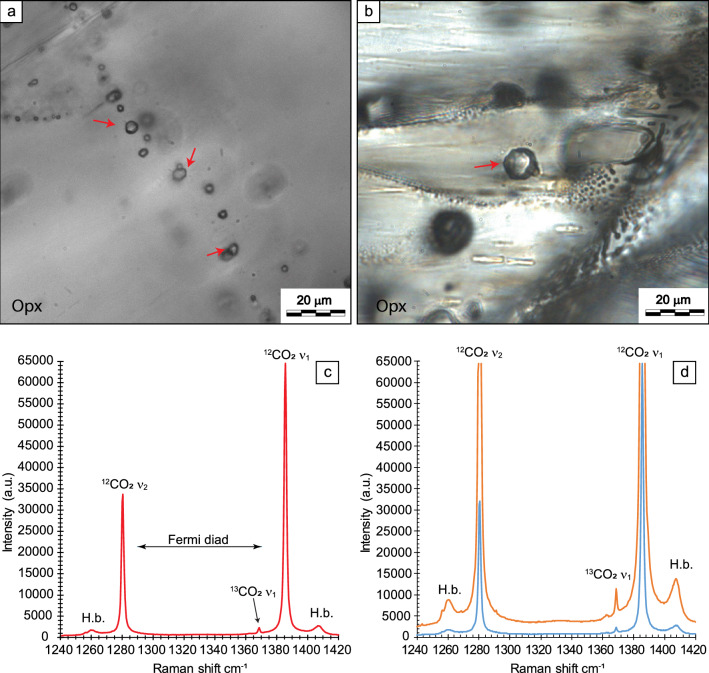
Microphotographs of selected fluid inclusions and CO_2_ Raman spectra collected during S.S. and D.S. analyses. (**a**,**b**) Microphotographs showing a secondary trail of fluid inclusions (F.I.) trapped in Opx and a primary fluid inclusion with negative crystal shape trapped in Opx in mantle rocks from El Hierro (Canary Islands) (red arrows indicate fluid inclusions selected for Raman analysis). (**c**) Raman spectrum of CO_2_ in a fluid inclusion (sample XML6_Fi3a). The two strong bands (upper ^12^CO_2_ ν_1_ and lower ^12^CO_2_ ν_2_ bands) at 1285 and 1388 cm^−1^ at ambient conditions, forming the Fermi diad, arise from the anharmonic mixing of the overtone of the symmetric bending mode 2ν_2_ with the symmetric stretching mode ν_1_ (Fermi resonance effect^43^). The ^13^CO_2_ upper band (ν_1_) composing the Fermi diad of the ^13^CO_2_ molecule is also present at about 1370 cm^−1^. The ^13^CO_2_ lower band is predicted at 1260 cm^−1^, but its actual frequency remains uncertain because it overlaps the more intense hot band, with a frequency at 1264 cm^−1^^28,31,61^. (**d**) CO_2_ Raman spectra of one selected fluid inclusion (sample XML11_Fi20), collected by single spectra (blue spectrum; acquisition time of 85 s; S.S.) and distinct spectra (D.S.) analyses (orange spectrum; acquisition time of 425 s). *Opx* orthopyroxene, *a.u.* arbitrary units, *H.b.* hot bands, *cm*^*−1*^ Raman shift.

Carbon stable isotope compositions of CO_2_ in the geosciences and beyond are critical for enabling studies on the nature and origin of fluids, Earth's degassing, and the geological cycle of carbon^1–5^. This is because relative differences in ^13^CO_2_/^12^CO_2_ isotope ratios discriminate the different Earth carbon reservoirs and their mixtures owing to isotopic fractionation^6–9^. At crustal to mantle depths, geogenic CO_2_ can be trapped (and preserved) in fluid inclusions, micrometre-sized cavities in rock minerals containing micro- to pico-moles of fluid (Fig. 1a,b)^10–13^. Their ^13^CO_2_/^12^CO_2_ ratio is expressed in delta (δ) notation^14^ and computed relative to an international measurement standard (the Vienna Pee Dee Belemnite (VPDB) standard) in per mil (δ^13^C_CO2_ ‰).

The carbon isotopic composition of Earth's deep CO_2_ is traditionally measured by bulk analyses of volcanic gas and fluid inclusions in mineral and rock samples using mass spectrometry, which meets requirements of measurement accuracy and precision but is also technically challenging due to the small quantity of fluid extracted from the crushing or heating of samples, the relatively large amount of fluid sample required, and the specific sample preparation^15,16^.

Raman micro-spectroscopy can also determine the δ^13^C_CO2_ with several advantages. First, it is possible to analyse in-situ 10^–6^–10^–8^ mol of CO_2_ in individual fluid inclusions of 10–20 µm in size or less^17–20^. Also, spectroscopic analyses are real-time, non-destructive and require minimal sample preparation, allowing for measuring carbon isotope signatures at the micrometre scale; such spatial resolution is impossible with bulk techniques.

Raman vibrational modes allow distinguishing the ^13^CO_2_ and ^12^CO_2_ isotopologue bands in spectra (Fig. 1c)^21,22^. Band intensities and areas are directly proportional to the number of corresponding vibrations in the sample volume and, consequently, linearly related to the relative concentrations of individual chemical species^23–25^. Pioneering studies^25,26^ were the first to apply this *quasi-*Lambert–Beer law to calculate the carbon isotopic composition (δ^13^C_CO2_) of CO_2_ in individual fluid inclusions based on ^13^CO_2_/^12^CO_2_ band area ratios. These authors reported very low reproducibility due to instrumental factors. More recently, a new generation of high-resolution confocal Raman systems and detectors renewed interest in in-situ analyses of ^13^CO_2_/^12^CO_2_ ratios in fluid inclusions and optical cells^27–33^. This research improved the analytical potential of Raman micro-spectroscopy in determining the carbon stable isotope composition of CO_2_. However, none of these studies matched precision and/or accuracy adequately, raising the question of the applicability of this technique for C stable isotopes punctual analysis of CO_2_ fluids.

The assumption of a linear relationship between the relative concentration of a molecular species and its contribution to the Raman spectrum is often not applicable. Instrumental drifts can induce subtle variations in the signal-to-noise ratio, affecting spectral output and, consequently, band intensity and shape. In order to reduce the intrinsic analytical errors related to the discrete nature of light photons (e.g., intensity fluctuations and different sources of noise), in this study, we developed experimental parameters for Raman measurements of stable carbon isotopes in individual CO_2_ fluid inclusions. We focused on high-density CO_2_ fluid inclusions in mantle rocks from two different localities—The Canary Islands and the Ethiopian plateau—to investigate the isotope signature of deep Earth fluids. We resolve analytical issues and present new carbon isotope ratios of CO_2_ in single fluid inclusions with an error better than 2.5‰ (all errors are 1σ unless stated otherwise), providing a method to effectively measure the mass ratio of carbon isotopes at the micrometre scale in geological samples.

## Theoretical and experimental rationale

### *Raman theory for C isotope ratio measurements of CO*_*2*_

Experiments described here for C isotope ratio measurements of CO_2_ were designed to be consistent with theoretical aspects of Raman spectroscopy. Quantitative Raman analyses in gas/fluid mixtures are based on Placzek's polarizability theory, which states that in a system of freely oriented molecules, the Raman scattering intensity depends on the number of scattering molecules within the system or analytical volume^23,24^. Thus, the Raman signal intensity is proportional to the component concentration according to:

1 $${I}_{i}=LI*K*P* {\sigma }_{i}*{X}_{i}$$

where I_i_ is the Raman signal intensity of the gas component i, LI is the laser intensity, K represents spectroscopic and analytical factors (i.e., the inherent Raman scattering efficiency of a molecule, the molecular interactions, the wavelength-dependent efficiency of the instrument, and the external environmental conditions^34–37^), P is the optical path length, σ_i_ is the Raman scattering cross-section of component i, and X_i_ is the relative amount (mol %) of component i^38^.

As shown by Eq. (1), band intensities are sensitive to laser power, molecular interaction (e.g., fluid composition and density/pressure), optics, and other analytical factors that are difficult to assess. Relating Raman band intensity with bandwidth through the *"real band intensity"*, defined as the product of the measured band intensity and full width at half maximum (FWHM), can reduce the uncertainties related to intensity count measurements^34^. This relation can be expressed with integrated band areas^34,37^, constraining the band intensity to the band shape in two-dimensional space^34,37^.

Therefore, for quantitative analyses in a gas mixture, the integrated band area, A_i,_ of single component i is proportional to its relative concentration, X_i_ (e.g., mole %)^17,18,39,40^ as follows:

2 $$ X_{i} = \frac{{\left[ {\frac{{A_{i} }}{{\left( {\sigma_{i} *\zeta_{i} } \right)}}} \right]}}{{\Sigma \left[ {\frac{{A_{n} }}{{\left( {\sigma_{n} *\zeta_{n} } \right)}}} \right]}} $$

where A_*i*_, σ_*i*,_ and ζ_*i*_ are the integrated band area, the wavelength-dependent Raman scattering cross-section, and the instrumental efficiency for species i, respectively; A_n_, σ_n_, and ζ_n_ are the band areas, the wavelength-dependent Raman scattering cross-sections, and the instrumental sensitivity, respectively, for all n species within the system. Because there is no significant variation in bond energy between ^13^CO_2_ and ^12^CO_2_ isotopologue molecules, as evidenced by the very close band position, we assume equal Raman scattering factors for isotopically substituted molecules^41^. In addition, the instrument sensitivity does not vary in the measured interval from 1200 to 1400 cm^−1^ (cf., “Methods” section). Consequently, the ^13^CO_2_ and ^12^CO_2_ integrated band area ratios express carbon stable isotopic mass ratios as δ^13^C_CO2_‰ notation according to the equation:

3 $${\delta }^{13}{C}_{CO2}=\left\{\left[\frac{{\left(\frac{{A}_{13CO2}}{{A}_{12CO2}}\right)}_{sample}}{{\left(\frac{{X}_{13CO2}}{{X}_{12CO2}}\right)}_{PDB}}\right]-1\right\}*1000$$

where ${\left(\frac{{X}_{13CO2}}{{X}_{12CO2}}\right)}_{PDB}$ is the carbon isotopic ratio of the standard Vienna Pee Dee Belemnite.

### *Raman spectroscopy of *^*12*^*CO*_*2*_* and *^*13*^*CO*_*2*_* isotopologues*

In the Raman spectrum of CO_2_, the ^12^CO_2_ and ^13^CO_2_ isotopologue molecules are recognised. As shown in Fig. 1c, two strong bands, referred to as the ^12^CO_2_ upper band (ν_1_) and ^12^CO_2_ lower band (ν_2_), respectively^42^, form the Fermi diad^43^; two hot bands flank the Fermi diad, at higher and lower wavenumbers, respectively compared to ν_1_ and ν_2_. A weak band to the left of the ^12^CO_2_ ν_1_ is the ^13^CO_2_ upper band (ν_1_)^44,45^. Because the heavier ^13^CO_2_ isotope is scarce compared to the more abundant ^12^CO_2_, the intensity and area of the ^13^CO_2_ band are about 10^2^ times weaker than the ^12^CO_2_ band (Fig. 1c). Relative differences in isotope ratios are presented in per-mil notation (‰) and precision at the fourth decimal unit is required^9^.

To improve the sensitivity of Raman CO_2_ isotopic analysis, we enhanced the signal-to-noise ratio by increasing the intensity of the exciting radiation in the scattering volume using a laser source with high power output (150 mW) and applying high confocality (100 µm pinhole) while using relatively short acquisition times (cf., Method Section). Long accumulation times—up to several hours—have been previously applied to enhance the intensity of the ^13^CO_2_ band, integrating the ^13^CO_2_ and the ^12^CO_2_ band areas measured in several consecutive accumulations^27–31^. However, extended accumulation times may induce spectral variations caused by instrumental drift, measurement errors or other artefacts, including fluorescence background and other sources of noise^46–48^ (cf., Supplementary Information Sect. S1), which affect baseline and band shapes and, consequently, the precision of band fitting. The proposed approach, however, does not overcome erratic analytical noise effects during analyses. To further improve the signal-to-noise ratio, we replicated spectra thrice in a statistically representative number of fluid inclusions in different mineral phases and performed spectra processing (cf., Supplementary Information Sect. S2).

## Results

### *CO*_*2*_* spectral analysis*

We analysed 42 CO_2_ fluid inclusions in olivine (Ol), orthopyroxene (Opx), and clinopyroxene (Cpx) from mantle rocks (Fig. 1a,b) from two different localities: Injibara, Lake Tana region in the Ethiopian Plateau (Ethiopia^49,50^) and El Hierro Island (Canary Islands, Spain^51^), which are part of a collection at the Università Milano Bicocca (cf., Supplementary Information Sect. S3). Twenty fluid inclusions were selected in Ol and Opx in mantle peridotites from the Ethiopian Plateau and twenty-two in Ol, Opx and Cpx from El Hierro Island. Inclusions have comparable sizes (5–21 µm) and depths within the samples (5–19 µm). We selected inclusions with high CO_2_ density (0.73–1.07 g/cm^3^) to increase the fluid mass in the analytical volume to a few micromoles (cf., Supplementary Information Sect. S3). We checked for other trace gas components (i.e., N_2_, CH_4_, H_2_S, H_2_O, SO_2_, CO) because these can modify the background, position, and shape of the CO_2_ bands^52^. Fluid inclusion data are summarised in Supplementary Table S1.

In each of the forty-two pure CO_2_ fluid inclusions, we collected two consecutive sequences of three Raman spectra at the same focal point (84 sets of 3 analyses for a total of 252 spectra) (Supplementary Table S2). For each fluid inclusion, in the first set of three spectra, accumulation times ranging from 35 to 360 s allowed simultaneous collection of ^13^CO_2_ and ^12^CO_2_ bands (single spectrum; S.S.) (^12^CO_2_ band intensities set at ≤ 60,000 counts; Fig. 1d). In the second set of three spectra, slightly longer accumulation times (175–1500 s) were used to enhance ^13^CO_2_ band intensity (cf., Method Section). With this instrumental setting, ^13^CO_2_ and ^12^CO_2_ bands were collected in two separate spectra (Fig. 1d; double spectra; D.S.).

**Figure 2:**
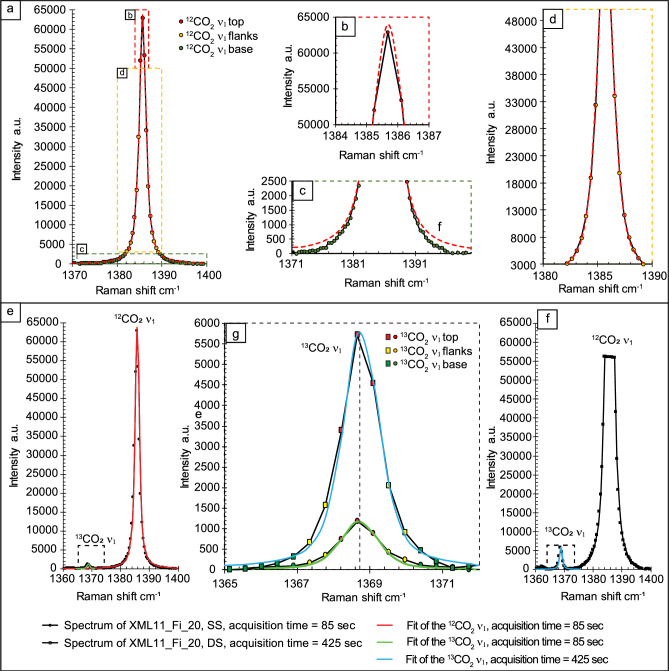
The fitting procedure adopted for spectral processing of the ^12^CO_2_ and ^13^CO_2_ bands. With the adopted Raman experimental protocol, the ^12^CO_2_ ν_1_ upper band is defined by 67 Raman sampling points, while the ^13^CO_2_ ν_1_ band by 13 sampling points for both S.S. and D.S. sets of measurements. (**a**) Example of fitting of the ^12^CO_2_ ν_1_ isotopologue. The enlargements on the top (**b**), the flanks (**c**) and the base (**d**) of the band show how chosen fitting curve and the fitting procedure model these three regions of the band. (**e**) Example of fitting of the ^13^CO_2_ ν1. (**e**) and (**f**) Examples of fitting the ^13^CO_2_ ν1 isotopologue in S.S. and D.S. analyses, respectively. The enlargement of the ^13^CO_2_ band (**g**) compares the adopted fitting procedure in S.S. (green fitted band) and D.S. (light-blue fitted band) analyses in the same fluid inclusion. Note that the fitting of the ^13^CO_2_ band resulting from D.S. longer accumulations is less accurate, slightly overestimating the integrated band area.

To improve the interpretation of Raman band parameters such as integrated position, shape and area, we performed spectral processing^37,47^, including baseline correction and spectral fitting, using the freeware software Fytik 1.3.1^53^. We manually removed baselines based on a least-squares method. Despite the automated baseline correction eliminating potential biases induced by the operator, it can generate secondary or external noise sources by producing new functions that oscillate around the real background value^47^. For each Raman spectrum, we specified the points of the spectral background to be removed^46^. We removed the base of ^13^CO_2_ ν_1_ and ^12^CO_2_ ν_1_ bands, where a change in the polarity of the band was observed at 1365 ± 0.2 and 1372 ± 0.2 cm^−1^ (^13^CO_2_ band), and 1371 ± 0.03 and 1399 ± 0.03 cm^−1^ (^12^CO_2_ band). Following baseline correction, we performed band fitting by Pseudo-Voigtian curves to obtain the most accurate band position, intensity, shape, and area^37^. We adopted a Split Pseudo-Voigt algorithm^53^ as the best statistical interpolator of ^12^CO_2_ and ^13^CO_2_ raw bands, correcting the apparent asymmetries of the two bands by modifying curve parameters such as the shape of the base, the full width at half maximum (FWHM), and the intensity (cf., Supplementary Information Sect. S2). As illustrated in Fig. 2, we interpolated the Split-Pseudo Voight curves with all the sampling points corresponding to the top, the flanks and the base of the ^12^CO_2_ and ^13^CO_2_ bands to achieve excellent fitting (i.e., R^2^ > 99). In some spectra, the error of the fitting algorithms for the considerably lower intensity ^13^CO_2_ bands increases, resulting in under- or over-estimations of the integrated band areas (Fig. 2f).

### *Calculation of *^*13*^*C*_*CO2*_*/*^*12*^*C*_*CO2*_* fitted area ratios*

**Figure 3:**
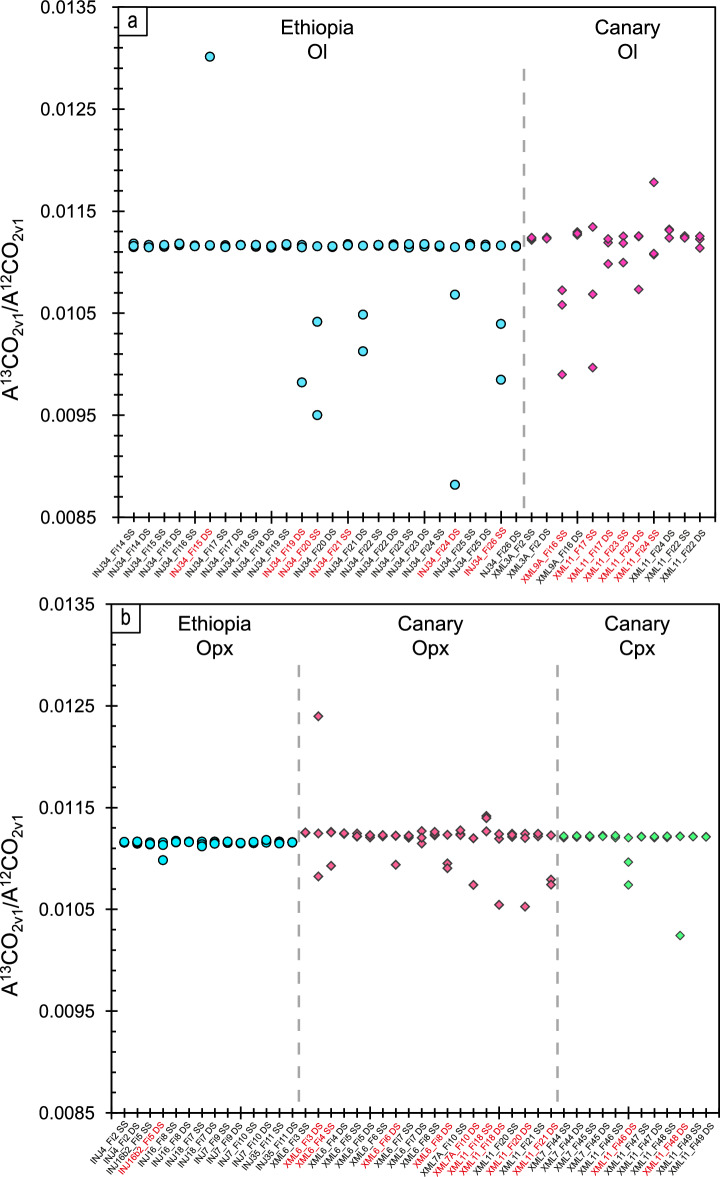
^13^CO_2_/^12^CO_2_ area ratios distribution calculated for each set of three spectra. Variation of the three area ratios calculated for single fluid inclusions trapped in Ol (**a**) and Opx and Cpx (**b**) in mantle rocks from Injibara (Lake Tana region, Ethiopia; circles) and El Hierro (Canary Islands; diamonds). The label tics distinguish between single spectra (S.S.) and distinct spectra (D.S.) sets of 3 analyses. Fifty-eight out of 84 sets of analyses are characterised by area ratios differing no more than 0.00005, while twenty-six sets of spectra (label tics in red) show at least one ^13^CO_2_/^12^CO_2_ band area ratio that differs by more than one order of magnitude from the others (from 0.00015 to 0.00233). These last sets of spectra (31% of the total) were found to be non-reproducible, so they were excluded from further analysis. *Ol* olivine, *Opx* orthopyroxene, *Cpx* clinopyroxene.

We calculated the averaged ^13^C_CO2_/^12^C_CO2_ integrated band area and area ratios for each set of three spectra and the standard deviation (1σ). Results are shown in Fig. 3 and reported in Supplementary Table S2. In fifty-eight sets of spectra out of eighty-four, corresponding to thirty-nine fluid inclusions, the three integrated ^13^CO_2_/^12^CO_2_ band area ratios were very consistent, with maximum variations in band area ratios ranging from 0.000003 to 0.000046. Conversely, in twenty-six sets of spectra, at least one ^13^CO_2_/^12^CO_2_ band area ratio differed by one or two orders of magnitude from the other two ratios (from 0.00012 to 0.00233; Fig. 3). These latter sets of spectra (31% of the total) were found to be non-reproducible for erratic analytical noise effects during analyses, and we excluded them from further analysis.

Outliers are mostly from longer D.S. analyses and suggest that the influence of instrumental effects on spectral fitting is amplified during longer analyses. We note from Fig. 3 that in most discarded analyses, the non-reproducibility of a single set of three integrated ^13^CO_2_/^12^CO_2_ band area ratios can be correlated with an underestimation of the ^13^CO_2_ band area, probably related to the relatively lower intensity of these bands above background, leading to a more significant error in determining the band area.

We further explored a possible dependence of average integrated ^13^CO_2_/^12^CO_2_ band area ratios on fluid density. No correlation has been observed in the explored range of fluid densities (Supplementary Fig. S1), confirming that density variations equally reflect on ^13^CO_2_ and ^12^CO_2_ band shapes and, consequently, integrated areas.

The precision of the fifty-eight averaged integrated ^13^CO_2_/^12^CO_2_ band area ratios measurements was tested in terms of the *reproducibility coefficient in per mil (RC‰),* calculated after Marshall et al.^27^:

4 $$RC\permil = \left[\frac{{1\sigma }_{\left(\frac{{A}_{13CO2\nu 1}}{{A}_{12CO2\nu 1}}\right)}}{{ Average}_{\left(\frac{{A}_{13CO2\nu 1}}{{A}_{12CO2\nu 1}}\right)}}\right]*1000$$

where ${1\sigma }_{\left(\frac{{A}_{13CO2\nu 1}}{{A}_{12CO2\nu 1}}\right)}$ is the averaged integrated area ratio standard deviation and ${Average}_{\left(\frac{{A}_{13CO2\nu 1}}{{A}_{12CO2\nu 1}}\right)}$ is the integrated area ratio average. Calculated RC*‰* range between 0.14 and 4.03‰, indicating excellent reproducibility (Supplementary Table S3)*.* As expected, reproducibility is better on average in those spectra where the ^13^CO_2_ and ^12^CO_2_ bands were collected simultaneously. Also, averaged integrated area ratios in fluid inclusions from Ethiopia show better reproducibility from 0.15 to 1.14‰ than fluid inclusions from the Canary Islands (RC from 0.14 to 4.03‰), independent from fluid inclusion size, density and depth within the sample (cf., Supplementary Table S1). This result outlines the contribution of the sample properties to the optical efficiency of the Raman set-up. In the present study, samples are thick (100–150 µm) transparent slices of rocks consisting of the same mineral phases. Thus, slight variations in spectral features are explainable by minerals' properties (e.g. presence of microfractures), impurities and other undiscovered noise sources (cf., Supplementary Information Sect. S1).

### Stable carbon isotopic composition (δ^13^C_CO2_‰) of individual CO_2_ fluid inclusions

**Figure 4:**
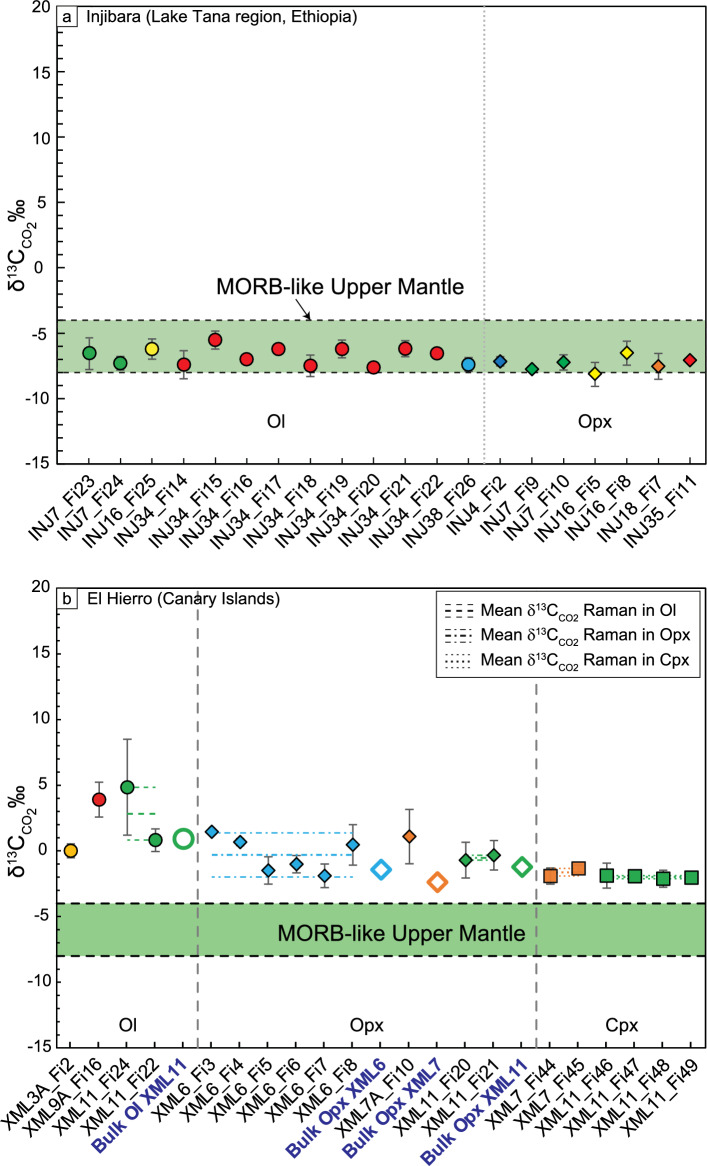
Raman-calculated δ^13^C_CO2_ values for CO_2_ fluid inclusions trapped in Ol (circles), Opx (diamonds) and Cpx (triangles) in peridotite samples from (**a**) the Lake Tana region (Ethiopia), and (**b**) El Hierro (Canary Islands). Analysed inclusions are divided by sample and are provided with error bars. The thick horizontal dashed black lines additionally observable for El Hierro measurements represent the mean bulk δ^13^C_CO2_ values obtained by isotope ratio mass spectrometry for comparison. The thin, dotted black lines represent the error interval for bulk Ol and Opx for El Hierro. The green field delimitates the "MORB-like Upper Mantle" carbon isotopic range (− 8‰ < δ^13^C < − 4‰^54^).

We calculated the stable carbon isotopic composition (δ^13^C_CO2_‰) of individual CO_2_ fluid inclusions based on the averaged integrated ^13^CO_2_/^12^CO_2_ area ratios by applying Eq. 3. The δ^13^C_CO2_values for mantle rocks of Lake Tana region (Ethiopia) and El Hierro (Canary Islands) are reported in Table 1 and Fig. 4. In mantle rocks from Ethiopia, olivine δ^13^C_CO2_ in thirteen fluid inclusions from four distinct samples ranges from − 7.60 to − 5.53‰ (mean = − 6.73 ± 0.66‰). In orthopyroxene, δ^13^C_CO2_ in seven fluid inclusions from five distinct samples ranges from − 8.16 to − 6.53‰ (mean = − 7.34 ± 0.52‰). Both olivine and orthopyroxene fluid inclusions show a substantial homogeneity in the isotopic composition of carbon (Fig. 4a) with similar standard deviations and δ^13^C_CO2_ values that fall within the expected CO_2_ upper mantle range (from − 8 to − 4‰^54^).

**Table 1:** δ^13^C_CO2_‰ calculated using Raman micro-spectroscopy in fluid inclusions trapped in Ol, Opx, and Cpx in mantle xenoliths from Injibara (Lake Tana region, Ethiopia) and El Hierro (Canary Islands).

	Host	Sample	F.I. analyses	δ^13^C_CO2_ Min	δ^13^C_CO2_ Max	δ^13^C_CO2_ Average/R.D	1σ
			n°	‰	‰	‰	‰
Injibara	Ol	INJ34	9	− 7.6	− 5.53	− 6.69	0.68
	Ol	INJ7	2	− 7.29	− 6.42	–	–
	Ol	INJ16	1	–	–	− 6.22	–
	Ol	INJ38	1	–	–	− 7.38	–
	Tot. Ol		13	− 7.6	− 5.53	− 6.73	0.66
	Opx	INJ4	1	–	–	− 7.15	–
	Opx	INJ7	2	− 7.74	− 7.24	–	–
	Opx	INJ16	2	− 8.16	− 6.53	–	–
	Opx	INJ18	1	–	–	− 7.53	–
	Opx	INJ35	1	–	–	− 7.06	–
	Tot. Opx		7	− 8.16	− 6.53	− 7.34	0.52
El Hierro	Ol	XML3	1	–	–	0.01	–
	Ol	XML9	1	–	–	3.91	–
	Ol	XML11	2	0.82	4.85	–	–
	Tot. Ol		4	− 0.01	4.85	2.4	2.34
	Opx	XML6	6	− 1.89	1.45	− 0.3	1.34
	Opx	XML7	1	–	–	1.09	
	Opx	XML11	3	− 0.71	− 0.34	− 0.52	0.26
	Tot. Opx		10	− 1.89	1.45	− 0.2	1.17
	Cpx	XML7	2	− 1.91	− 1.34	− 1.63	–
	Cpx	XML11	4	− 2.12	− 1.89	− 1.99	0.09
	Tot. Cpx		6	− 2.12	− 1.34	− 1.87	0.27

Reported δ^13^C_CO2_ values correspond to the isotopic ratios calculated for the fluid inclusions belonging to each sample within the same host mineral.

Raw data (R.D.) indicate the isotopic ratio calculated for samples where only a single fluid inclusion was analysed.

*Ol* olivine, *Opx* orthopyroxene, *Cpx* clinopyroxene, *n°* number, *Min* minimum, *Max* maximum.

In mantle rocks from the Canary Islands, olivine δ^13^C_CO2_ in four fluid inclusions from three distinct samples ranges from 0.01 to 4.85‰ (mean = 2.40 ± 2.34‰). In orthopyroxene, δ^13^C_CO2_ in ten fluid inclusions from three distinct samples ranges from -1.89 to 1.45‰ (mean = − 0.20 ± 1.17‰). In clinopyroxene, δ^13^C_CO2_ in six fluid inclusions from two distinct samples ranges from − 2.12 to − 1.34‰ (mean = − 1.87 ± 0.27‰). There is a progressive decrease of δ^13^C_CO2_ values and relative standard deviations from olivine to orthopyroxene and clinopyroxene fluid inclusions, the latter showing the highest homogeneity in the measured ratios (Fig. 4b). In general, the δ^13^C_CO2_ values measured in mantle rocks from El Hierro Island fall outside (well above) the expected CO_2_ upper mantle range, overlapping the range of values reported for limestone (from − 1 to + 1‰^2^).

## Discussion

The present results confirm that Raman microspectroscopy is reliable for measuring stable carbon isotopes of CO_2_ in individual fluid inclusions. The advantage of this method is that it is non-destructive and spatially resolved at the micron scale, which reveals a promising application prospect for the analysis of geogenic CO_2_ fluids. We succeeded in performing isotope measurements by developing a simple strategy for improving spectral analysis, thus reducing erratic analytical noise effects. High laser power, high confocality, and short acquisition times should ensure the optics' best efficiency to collect the highest signal-to-noise ratio to successfully investigate δ^13^C_CO2_ in a single CO_2_ fluid inclusion. With this novel analytical configuration, most analyses are characterised by high reproducibility (RC = 0.14–4.03‰), allowing us to calculate δ^13^C_CO2_ with 1σ better than 2.5‰. Noteworthy, δ^13^C_CO2_ determinations expressed as integrated and averaged (i.e., three consecutive analyses) area ratios and the simultaneous collection of ^13^CO_2_ and ^12^CO_2_ scattering cancel most uncertainties related to instrumental performance without applying instrument corrections.

Without reliable reference standards of known isotopic composition, we gauged the accuracy of Raman δ^13^C_CO2_ measurements by comparing present results with those obtained by isotope-ratio mass-spectrometry technique in the same rock samples. We point out that the latter technique implies that the obtained δ^13^C_CO2_ signature refers to the bulk of fluid inclusions hosted in the analysed crystals. Carbon stable isotope measurements by bulk mass spectrometry in minerals from four rock samples from the Canary Islands report δ^13^C_CO2_ values averaging 0.38‰ in Ol, − 1.74‰ in Opx and − 1.94‰ in Cpx (1σ =  ± 0.3‰^55^). As shown in Fig. 4b, there is a good agreement among the δ^13^C_CO2_ values reported in the different samples and minerals analysed with the Raman and conventional ratio mass spectrometric techniques. Notably, the inter-sample variability of the δ^13^C values is similar for both methods. Raman calculated δ^13^C_CO2_ values, although slightly heavier, fall in the same intervals (Fig. 4b). As an example, in Ol (sample XML11), the Raman mean δ^13^C_CO2_ value is 2.83 ± 2.01‰ (Table 1), while mass spectrometry analyses calculate δ^13^C_CO2_ 0.96 ± 0.30‰.

Similarly, in Opx (samples XML6, XML7 and XML11), the average Raman δ^13^C_CO2_ values from fluid inclusions are − 0.30 ± 1.34‰, 1.09 ± 2.16‰ and − 0.52 ± 0.26‰, respectively (Table 1), whereas bulk mass analyses of the same rock samples indicate − 1.43‰, − 2.38‰ and − 1.23‰ (Fig. 4), respectively^55^. The 1σ δ^13^C_CO2_ values from ± 0.26 to ± 2.16‰ obtained with Raman are, in most cases, higher compared to those of conventional mass spectrometry in bulk fluid inclusions (1σ δ^13^C_CO2_ =  ± 0.30‰^56,57^). Nevertheless, Raman-based carbon stable isotope calculations are accurate and precise enough to record the slight variations of δ^13^C_CO2_‰ in the different minerals. To account for enriched ^13^C observed in mantle CO_2_ fluids from the Canary Islands, Sandoval-Velasquez et al.^55^ suggested a recycled crustal carbon component in the El Hierro mantle source, previously unidentified in volcanic gases/groundwater studies in the region. It is beyond the scope of this study to consider the scientific implications of the obtained results. Here, we focus on the reliability of the carbon isotopic measurements obtained in fluid inclusions of mantle rocks through the Raman technique. The present study suggests that Raman microspectroscopy can be an integrative method for δ^13^C_CO2_‰ determination in geological investigations. It gives a new perspective approach to push C isotopic measurement to the micrometre scale and enables applications such as tracing the origin of different CO_2_ fluid fluxes within the Earth.

## Methods

### Raman microspectroscopy

CO_2_ Raman spectra have been collected on thick (100–150 µm) rock sections polished on both sides by the HORIBA LabRAM HR Evolution Raman System at the Dipartimento di Scienze dell'Ambiente e della Terra (DISAT), Università di Milano-Bicocca. The spectrometer system has an 800 mm focal distance and is coupled with an air-cooled 1024 × 256 px CCD detector cooled by Peltier effect (− 70 °C). Single point analyses have been performed using a linearly polarised solid-state green laser source at 532.06 nm with a nominal 300 mW output, powered at 150 mW by the 50% neutral density filter. Raman spectra acquisition was performed with a backscattered geometry by focusing the laser beam inside fluid inclusions to a maximum depth of 20 µm below the sample surface using a transmitted light Olympus BX41 microscope. A × 100 objective (numerical aperture [N.A.] = 0.90) with a long working distance was used for all the acquisitions to increase spatial resolution (≤ 1 µm^3^). The confocal pinhole was set at 100 µm diameter. The 1800 grooves per mm grating allow a spectral interval coverage from 1069.98 to 1522.70 cm^−1^ with a spectral per pixel resolution of about 0.44 cm^−1^/px. Accumulation times were varied from 30 s to 8 min to achieve suitable signal-to-noise enhancement. Each measurement was repeated thrice at the conditions for statistical analyses. Calibration was performed daily based on the auto-calibration process by the Raman system Service relative to the zero line and the silicon standard (520.7 cm^−1^), according to the ASTM 1840-96 normative^58,59^. The linearity of the spectrometer was also automatically checked and corrected during the process^60^. The spectrometer efficiency in the considered wavenumber region was checked with a white lamp of known emission intensity. A further considered point is the effect of temperature variation on grating dispersion and spectrometer focal length. Therefore, the temperature in the laboratory was pre-set at 20 °C and maintained constant within ± 0.5 °C.

### Supplementary Information

Supplementary Information 1.

Supplementary Tables.

## Data Availability

All data needed to evaluate the conclusions are in the paper and the Supplementary Information.
