# Supplementary material for: Spatially resolved CO2 carbon stable isotope analyses at the microscale using Raman spectroscopy
Source: Sci Rep. 2023 Oct 29;13:18561. doi: 10.1038/s41598-023-44903-z (PMC10613625; doi:10.1038/s41598-023-44903-z)
Supplement: Supplementary file 1 — Supplementary Information 1. [file 41598_2023_44903_MOESM1_ESM.docx]

**Supplementary Information**

***Figure S.1.*** *Density variations in CO_2_ fluid inclusions compared with calculated δ^13^C_CO2_ values. Only CO_2_ of high density (0.73 to 1.07 g/cm^3^) were selected to increase the fluid mass to a few micromoles. At the top of the plot, we report fluid inclusions in samples from El Hierro, while at the bottom, those in Injibara.*

1. **Optimising Raman sensitivity for quantitative analyses**

The application of Raman spectroscopy for calculating the ^13^C_CO2_ /^12^C_CO2_ concentration ratio from spectra requires high-precision measurements. Many factors influence the precision with which band positions and areas can be measured. Those considered in the present study are reported below.

*1.1 Spectral resolution*

The precision with which band positions and areas are measured is closely related to the intensity and the width of the considered bands, defining their shape^1^. This latter, in the first place, is determined by the spectral resolution, defined as the ability of the spectrometer to separate two neighbouring Raman lines, λ and λ+Δλ ^2^. Spectral resolution determines the magnitude of measured Raman shifts per pixel in wavenumbers (cm^-1^/px; i.e., in the maximum number of Raman shifts recorded within a single pixel or the laser dispersion) and contributes to the definition of the graphical shape of the band in terms of the number of points sampled per cm^-1^. Spectral resolution depends on factors which are instrument, experiment, and sample-specific. Instrument (e.g., optical path and the number of pixels of the detector) and sample (e.g., peak position and Raman cross-section) specific parameters cannot be modified. Thus, experiment-specific parameters, particularly the laser wavelength, the grating groove density and the entrance slit widths, should be set to improve spectral resolution (cf. analytical methods).

*1.2 Noise sources*

Noise sources affect the quality of spectra, mainly reflecting on the Raman signal intensity. The noise is defined as that part of the spectrum carrying unwanted information from a random fluctuation of the Raman signal intensities^3^. The noise ($\sigma_{y}$) is made up of five components, and it is expressed as [Eq.6] ^4^:

$\sigma_{y}= \left( \sigma_{S}^{2}+\sigma_{B}^{2}+\sigma_{d}^{2}+\sigma_{F}^{2}+\sigma_{r}^{2} \right)^{\frac{1}{2}}$ [Eq.6]

where $\sigma_{S}$ is the shot noise, $\sigma_{B}$ is the background noise, $\sigma_{d}$ is the dark signal noise, $\sigma_{F}$ is the flicker noise, and $\sigma_{r}$ is the readout noise.

Among these, the $\sigma_{S}$, the $\sigma_{B}$, the $\sigma_{d}$, and the $\sigma_{r}$ noises affect the resolution of the shape of the bands in Raman spectra:

- $\sigma_{S}$ (shot noise): is the result of the inconsistent intensity of photons collected per pixel per unit of time and is governed by a Poisson distribution. The shot noise is an unescapable random source of noise in Raman spectra and cannot be excluded from the signal^3^. The only way to minimise the effect of the shot noise is to collect a higher number of photons; this can be accomplished by increasing the slit width and the pinhole apertures, applying prolonged acquisition times, or increasing the laser power. However, the first two considered parameters are responsible for increasing other noise sources, such as the instrumental generated background noise or the dark signal noise^2,3,4,5^. For this reason, we applied a laser power higher than usual for analyses in geological samples while keeping a short accumulation time and small pinhole.
- $\sigma_{B}$ (background noise): is the random Raman band intensity variation caused by the sample (sample generated background noise, i.e., sample physical characteristics, fluorescence, thermal emissions^3,4,5^) and by the laser (instrumental generated background noise; i.e., thermal heating of the optics, laser line reflections due to the optics or due to dust on the samples^3,4,5^). In Raman spectra of fluid inclusions, the most commonly considered causes generating sample background noise effects are:

1. the host mineral: minerals having high refractive indices (i.e., garnet or olivine), or being fluorescent, contribute to reducing the signal-to-noise ratio of the Raman scattering from the fluid inclusion^3,6,7,8^;
2. Depth and habits of the analysed fluid inclusion: fluid inclusions with variable sizes and habits can be trapped at various depths within the host minerals. Fluid inclusions located at a depth of more than 20 microns within the sample and/or with irregular shapes would reduce the number of light photons which can successfully reach and excite the analysed CO_2_ molecules compared to the surrounding host mineral, influencing the collected scattered Raman radiation, thus, signal to noise ratio in the spectrum^2,6,9,10,11,12^;
3. presence of metastable phases within the fluid inclusions (i.e., the presence of bubbles in liquid fluid inclusions, which can move during the analyses due to the laser heating);
4. presence of gas mixtures: molecular interactions between two or more gas components can generate local order/disorders effects, which affect the strength of the chemical bonds of the molecules composing the system, modifying the shape of the Raman bands. This leads to incorrect band positions or areas measurements^3,13^;
5. the density of the fluid and temperature variations: as with the fluid inclusion composition, these factors can lead to variations in the strength of the molecular bonds and, in turn, the gas phase Raman scattering, leading to a decrease in the signal-to-noise ratio in the Raman spectrum^3,9,13,14,15^.

Thus, selecting shallow (close to the mineral surface), regularly-shaped inclusions containing pure CO_2_ increases Raman sensitivity. Similarly, reducing the pinhole and adopting large magnifications reduce the sample background noise. However, we should note that instrumental background noise effects cannot be eliminated from the Raman spectra^3,4,6,11^.

- $\sigma_{d}$ (dark signal noise): is the spontaneous thermal generation of electrons within the CCD detector during analyses. Changes in temperature can be recorded on the CCD detectors during the analyses during the laser heating^3,4,5^;

- ${}_{r}$ (readout noise): is the standard deviation associated with the digital conversion of scattered electrons from the detector to the spectrum. The readout noise is inherent in every signal acquisition, independent of the laser or the magnitude of the scattered radiation reaching the CCD detectors^3,4,5^.

1. **Raw spectra processing**

The uncertainties in the measurements of band positions and areas produced by the noise effects on the raw Raman spectra can be further mitigated by spectral processing. Raw spectra can be processed through baseline removal and band fitting^1,3^. The shape of the baseline is determined by the sum of all the noise components. Currently, automated baseline correction software is available to identify the points of the spectrum that belong to the Raman bands and fit the remaining ones with appropriate baseline functions^3,16^. These autocorrections, however, can be responsible for generating a secondary or external source of noise by producing new functions oscillating around the real background value they are trying to remove^3^. In many cases, manual baseline removal specifying those background points which should be equal to zero is preferred; however, manual baseline correction could be affected by bias introduced by operators, the function which is generated to fit the specified points to be removed is forcibly less sensitive to oscillations of the background values^4^.

CO_2_ spectra have been treated with baseline correction and band fitting with a Split Pseudo Voight function by the freeware software Fityk^17^ to perform precise measures of band positions and areas. CO_2_ symmetric stretching modes are characterised by asymmetric band profiles, resulting from the sum of Gaussian and Lorentzian components (i.e., Pseudo-Voigt behaviour^1,2^). The Gaussian component of the symmetric stretching profile increases with the increasing ratio of spectral resolution/FWHM, inducing an enlargement of the band at the base and a decrease in intensity at the peak maximum^2^. Symmetric CO_2_ bands, however, are further characterised by “apparent” asymmetries on the shapes of the bands due to the overlapping of the signals (e.g., ^13^CO_2_ ν_1_ and the ^12^CO_2_ ν_1_ ^18,19^), fitting by mean of a Pseudo-Voigt profile can lead to imprecise measurements. Fitting curves for asymmetric Raman bands should be used, such as the Pearson VII profile^1^. Many fitting programs implement a Split Pseudo-Voigt fitting algorithm which allows the processing of symmetric Raman bands affected by “apparent” asymmetries (e.g. Fityk^17^). These profiles can improve the accuracy of band parameters up to 30 times^1,3,17,20^. Asymmetries in the shapes of the CO_2_ bands depend on fluid density, and below the critical density (0.466 g/cm^3^), gas-like CO_2_ bands become rapidly narrower at decreasing density, significantly affecting profiles^21^. For this reason, spectral treatment of CO_2_ bands in fluids having densities below 0.2 g/cm^3^ (e.g., melt inclusion bubbles) does not reveal the genuine parameters of these bands^21,22^.

1. **Mantle rock petrography**

Fluid inclusions analysed for the present work are hosted in mantle peridotite xenoliths collected from El Hierro (Canary Islands) and Lake Tana (Ethiopia). The following paragraphs summarise mantle rocks and fluid inclusion petrography^11,12,23,24,25^.

### 3.1 El Hierro (Canary Islands)

El Hierro is the westernmost and youngest island of the Canary Islands archipelago^26^. Its sub-aerial magmatic activity started at approximately 1.12 Ma in the NE part of the island^27^, with the most recent eruption occurring in 2011-2012 in the La Restinga area. The magmatic activity is characterised by intraplate oceanic volcanism, dominated by alkali-basalts, minor tholeiites, and differentiated lavas. The mantle xenoliths considered for the present work have been collected from the El Julan Cliff Valley in massive lava flows dated at approximately 40-30 ka.

Peridotites consist of spinel-dunites (Ol: 92-94 vol%; Opx: 4-6 vol%; Cpx: 1-4 vol%), spinel-harzburgites (Ol: 59-78 vol%; Opx: 18-38 vol%; Cpx: 2-4 vol%), and spinel-lherzolites (Ol: 63-78 vol%; Opx: 11-26 vol%; Cpx: 11-12 vol%). Rocks are characterised by protogranular texture with variable recrystallisation degree (around 20% on average). Two generations of olivine (Ol) and orthopyroxene (Opx) have been recognised: Ol I and Opx I porphyroclasts (Fig. S.2a) consist of large (up to 10 mm) strained crystals, while Ol II and Opx II (Fig. S.2b) in strain-free interstitial grains or aggregates of polygonal grains with triple junctions. Opx I can contain exsolution lamellae of clinopyroxene (Cpx) + spinel (Spl), but in more recrystallised peridotites, it shows clear rims. Cpx and Spl are also present as small (1 mm on average; Fig. S.2) subhedral or interstitial grains. Olivines (Ol I and Ol II) are Fo-rich, with Mg-numbers (Mg# = Mg/(Mg + Fe_tot_)*100, molar) from 89 to 91, being higher in spinel-harzburgites. Orthopyroxenes (Opx I and Opx II) are En-rich, with Mg# similar to those of Ol I and II, from 90 to 91. Cpx is Cr-Diopside, with a wide Mg# range from 89 to 93. Spl is variable in composition, being a Mag-Spl solid solution, with Cr-number (Cr# = Cr/(Cr + Al)*100, molar) from 25 to 35. Moreover, some Spl grains are characterised by Chr-rich rims, having Cr# roughly from 40 to 50. Peridotites equilibrated at mantle conditions, between 1.5-2 Gpa, at 900-1100°C.


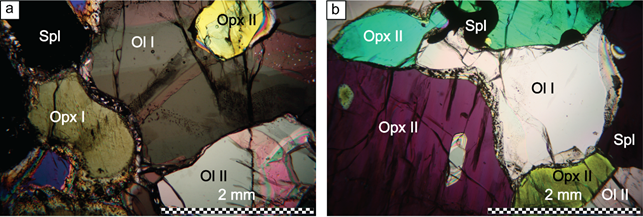


***Figure S.2*** *Microphotographs of peridotitic samples from El Hierro, Canary Islands. a) porphyroclasts of Ol I and Opx I, and Spl subhedral grains associated with neoblasts of Ol II and Opx II in an Spl-lherzolite (XML8); b) porphyroclasts of Ol I associated with neoblasts of Opx II and Spl subhedral grains in a Spl-harzburgite (XML11).*

Two different generations of fluid inclusions are present within all the rock-forming minerals. They are classified as *Early Type I* and *Late Type II*. *Early Type I* fluid inclusions show the association of mono-phase (L) CO_2_ ± N_2_ (N_2_ ≈ 0-18 mol%) inclusions (*Type 1a*; Fig. S.3a) coexisting with multiphase solid (≥ 70 vol% of daughter minerals) inclusions (*Type Ib),* consisting of Anh+ Sulfohalite+ Na-K chlorides+ Ap+ Dol+ Mg-Cal+ Spl+ Mgs+ Mg-sulf+ Tlc + CO_2_ ± N_2_ (N_2_ = 0.3-1.25 mol%)+ H_2_O (Fig. S.3b). Fluid inclusions density is up to 1.19 g/cm^3^. *Early Type I* fluid inclusions are often associated with intragranular and interstitial carbonate-silicate glass microveins. *Late Type II* fluid inclusions are found in secondary intergranular trails in all the mineral phases and consist of mono-phase (L) CO_2_, having densities varying from 0.37 to 1.08 g/cm^3^.


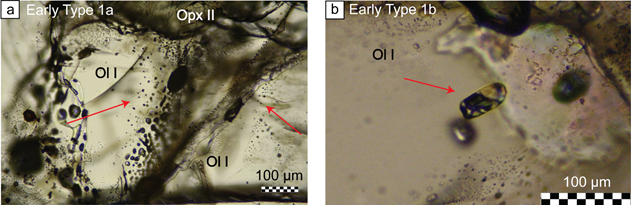


***Figure S.3*** *Microphotographs of studied fluid inclusions types in mantle peridotites from El Hierro (Canary Islands), modified after Remigi et al.^28^. a) Early Type 1a, CO_2_ + N_2_ and b) Early Type 1b multiphase solid fluid inclusions trapped in Ol porphyroclast (Ol 1).*

### 3.2 Lake Tana region (Ethiopia)

The Lake Tana region is located in the northwestern and recent part (Miocene-Quaternary) of the Ethiopian Plateau. The region belongs to the Miocene-Quaternary volcanic deposits of the Ethiopian Volcanic Province (EVP^29,30^). The Quaternary volcanic activity in the region developed within the Tana graben, and it is characterised by fissure-type lava fields and small- to medium-sized tuff cones, tuff rings and maars. Erupted volcanics are mainly porphyritic, vesicular alkali basanites with typical geochemical features of intraplate magmatism. The mantle xenoliths considered for the present work come from the Quaternary lavas, consisting of a massive, sub-aphyric and weakly vesiculated lava flow that erupted from a small scoria cone located SW of Injibara.

Mantle peridotites consist of spinel-lherzolites (Ol: 46-69 vol%; Opx: 19-31 vol%; Cpx: 9-22 vol%). Several samples show additional presence (up to 1 vol%) of Amp. *Spinel-lherzolites* are characterised by protogranular to porphyroclastic textures. Two generations of Ol and Opx have been recognised: Ol I and Opx I porphyroclasts consist of large (2-4 mm) deformed porphyroclasts, while Ol II and Opx II form polygonal neoblasts (up to 1 mm in size). Opx I contains Cpx-exsolution lamellae. Clinopyroxene is present as smaller (≈1 mm) interstitial and tabular grains and can contain Spl-exsolution lamellae. Spinel is porphyroclastic or holly-leaf shaped. Where present, amphibole occurs in contact with Cpx and contains relics of Spl. Olivine (Ol I and Ol II) are Fo-rich, with Mg# ranging from 89.2 to 89.6. Orthopyroxenes (Opx I and Opx II) are En-rich, with Mg# from 89.9 to 90.3. Cpx is Ti-poor, Cr-Na-rich diopside, with Mg# from 89.8 to 91. Spl has Mg# from 72.9 to 75.1 and Cr# from 15 to 19. Amp is Cr-rich Prg, Mg # from 87.5 to 88.2, and Cl-rich. Peridotites have been equilibrated at mantle conditions, between 1.3-2 Gpa, at 950-1150°C. Trace elements on Cpx showed LREE enrichment on HREE and flat REE patterns. HREE were relatively high, excluding re-equilibration with garnet. In particular, positive anomalies of LILE, in particular Th, U, Pb, and LILE/HFSE fractionation, were observed. Ti, Zr, and Hf showed moderate negative REE anomalies, and Nd and Ta contents were lower than those in the primitive mantle. Pargasite was Cl-rich and characterised by LREE, Pb, U, Th, and Sr, similar to Cpx, but it concentrated Ba. Fluid inclusion study

Fluid inclusions are present as small clusters and trails in Ol I and Opx I, and are rare in Cpx. In Ol I, few fluid inclusions contain CO_2_±H_2_O, while most react with the host mineral and are filled by daughter Tlc or Cal+Mgs without any fluid. In Opx I, fluid inclusions consist of CO_2_ ± H_2_O (CO_2_ ≥ 78-100 mole%). H_2_O has been detected by Raman micro-spectroscopy in a few fluid inclusions trapped in Ol I and Opx I. Eutectic temperatures measured in fluid inclusions indicated the additional presence of Na^+^, Mg^2+^ and Fe^2+^, with little or no Ca^+^ within the aqueous fluid. Fluid inclusions density resulted in being around 1.12 g/cm^3^.

### **References**

1. Yuan, X., Mayanovic, R. A. (2017). An empirical study on Raman peak fitting and its application to Raman quantitative research. *Applied Spectroscopy*, **71(10)**, 2325-2338; <https://doi.org/10.1177/0003702817721527>
2. Dubessy, J., Caumon, M. C., Rull, F., Sharma, S. (2012). Instrumentation in Raman spectroscopy: elementary theory and practice. *In* *Raman spectroscopy applied to earth sciences and cultural heritage*, **12**, 83-172.
3. Pelletier, M. J., (2003). Quantitative Analysis Using Raman Spectrometry. *Applied Spectroscopy*, **57**, 20A-42A; <https://www.osapublishing.org/as/abstract.cfm?URI=as-57-1-20A>
4. McCreery, R. L. (2000). Raman spectroscopy for chemical analysis. John Wiley and Sons, Vol. 225.
5. Barton, S. J., Kerr, L. t., Domijan, K., Hennelly, B. M. (2016). On the effect of experimental noise on the classification of biological samples using Raman micro-spectroscopy. *Proceedings, SPIE 9887, Biophotonics: Photonic Solutions for Better Health Care V*, **98873A**; <https://doi.org/10.1117/12.2227785>
6. Burke, E. A. (2001). Raman microspectrometry of fluid inclusions. *Lithos*, **55(1-4)**, 139-158; <https://doi.org/10.1016/S0024-4937(00)00043-8>
7. Caumon, M. C., Tarantola, A., and Mosser‐Ruck, R. (2015). Raman spectra of water in fluid inclusions: I. Effect of host mineral birefringence on salinity measurement. *Journal of Raman Spectroscopy*, **46(10)**, 969-976; <https://doi.org/10.1002/jrs.4708>
8. Caumon, M‐C, Tarantola, A, Wang, W. (2019). Raman spectra of gas mixtures in fluid inclusions: Effect of quartz birefringence on composition measurement. *Journal of Raman Spectrosc*opy, **51,** 1868– 1873; <https://doi.org/10.1002/jrs.5605>
9. Dubessy, J., Poty, B., Ramboz, C. (1989). Advances in COHNS fluid geochemistry based on micro-Raman spectrometric analysis of fluid inclusions. *European journal of Mineralogy*, **1(4)**, 517-534; <https://doi.org/10.1127/ejm/1/4/0517>
10. Fukura S, Mizukami T, Odake S, Kagi H. Factors Determining the Stability, Resolution, and Precision of a Conventional Raman Spectrometer. Applied Spectroscopy. 2006;60(8):946-950. doi:10.1366/000370206778062165
11. Frezzotti, M. L., Tecce, F., & Casagli, A. Raman spectroscopy for fluid inclusion analysis. J. of Geochem. Explor. 112, 1-20; https://doi.org/10.1016/j.gexplo.2011.09.009 (2012a).
12. Frezzotti, M. L., Ferrando, S., Tecce, F., and Castelli, D. (2012b). Water content and nature of solutes in shallow-mantle fluids from fluid inclusions. *Earth and Planetary Science Letters*, **351**, 70-83; <https://doi.org/10.1016/j.epsl.2012.07.023>
13. Le, V., Caumon, M., Tarantola, A., Randi, A., Robert, P., Mullis, J. (2019). Quantitative Measurements of Composition, Pressure, and Density of Microvolumes of CO₂–N₂ Gas Mixtures by Raman Spectroscopy. *Analytical chemistry,* **91**, 14359-14367; <https://doi.org/10.1021/acs.analchem.9b02803>
14. Seitz, J. C., Pasteris, J. D., & Chou, I.-M. (1996). Raman spectroscopic characterization of gas mixtures; II, Quantitative composition and pressure determination of the CO 2 -CH 4 system. American Journal of Science, 296(6), 577–600. <https://doi.org/10.2475/ajs.296.6.577>
15. Sublett, Jr D. M., Sendula, E., Lamadrid, H., Steele‐MacInnis, M., Spiekermann, G., Burruss, R. C., Bodnar R. J. (2019). Shift in the Raman symmetric stretching band of N_2_, CO_2_, and CH_4_ as a function of temperature, pressure, and density. *Journal of Raman Spectroscopy*, **51(3)**, 555-568; <https://doi.org/10.1002/jrs.5805>
16. Stellman, C. M., Aust, J. F., Myrick, M. L. (1995). In Situ Spectroscopic Study of Microwave Polymerization. *Applied Spectroscopy*, **49(3)**, 392–394; <https://doi.org/10.1366/0003702953963535>
17. Wojdyr, M. (2010). Fityk: a general‐purpose peak fitting program. *Journal of Applied Crystallography*, **43**, 1126-1128; <https://doi.org/10.1107/S0021889810030499>
18. Irmer, G., Graupner, T. (2002). Isotopes of C and O in CO_2_: a Raman study using gas standards and natural fluid inclusions. Acta Univ. Carolinae, Geol, 46, 35-36.
19. Hurai, V., Huraiová, M., Slobodník, M., Thomas, R. (2015). Geofluids: developments in microthermometry, spectroscopy, thermodynamics, and stable isotopes. 348 pp, Elsevier.
20. Weber, W. H., Zanini-Fisher, M., Pelletier, M. J. (1997). Using Raman Microscopy to Detect Leaks in Micromechanical Silicon Structures. *Applied Spectroscopy*, **51(1)*,*** 123–129; <https://doi.org/10.1366/0003702971938876>
21. Remigi S, Mancini T, Ferrando S, Frezzotti ML. Interlaboratory Application of Raman CO_2_ Densimeter Equations: Experimental Procedure and Statistical Analysis Using Bootstrapped Confidence Intervals. Applied Spectroscopy. 2021;75(7):867-881. doi:10.1177/0003702820987601
22. Lamadrid, H. M., Moore, L. R., Moncada, D., Rimstidt, J. D., Burruss, R. C., Bodnar, R. J. Reassessment of the Raman CO_2_ densimeter. Chem. Geol. 450, 210-222; https://doi.org/10.1016/j.chemgeo.2016.12.034 (2017).
23. Ferrando, S., Frezzotti, M. L., Neumann, E. R., De Astis, G., Peccerillo, A., Dereje, A., ... and Teklewold, A. (2008). Composition and thermal structure of the lithosphere beneath the Ethiopian plateau: evidence from mantle xenoliths in basanites, Injibara, Lake Tana Province. *Mineralogy and Petrology*, **93(1)**, 47-78; <https://doi.org/10.1007/s00710-007-0219-z>
24. Frezzotti, M. L., Ferrando, S., Peccerillo, A., Petrelli, M., Tecce, F., Perucchi, A. (2010). Chlorine-rich metasomatic H_2_O–CO_2_ fluids in amphibole-bearing peridotites from Injibara (Lake Tana region, Ethiopian plateau): nature and evolution of volatiles in the mantle of a region of continental flood basalts. *Geochimica et Cosmochimica Acta*, **74(10)**, 3023-3039; <https://doi.org/10.1016/j.gca.2010.02.007>
25. Oglialoro, E., Frezzotti, M. L., Ferrando, S., Tiraboschi, C., Principe, C., Groppelli, G., and Villa, I. M. (2017). Lithospheric magma dynamics beneath the El Hierro Volcano, Canary Islands: insights from fluid inclusions. *Bulletin of Volcanology*, **79(10)**, 1-17; <https://doi.org/10.1007/s00445-017-1152-6>
26. Becerril, L., Galve, J. P., Morales, J. M., Romero, C., Sánchez, N., Martí, J., and Galindo, I. (2016). Volcano-structure of El Hierro (Canary Islands). *Journal of Maps*, ***12*(sup1)**, 43-52; <https://doi.org/10.1080/17445647.2016.1157767>
27. Carracedo, J. C., Badiola, E. R., Guillou, H., de la Nuez, J., and Pérez Torrado, F. J. (2001). Geology and volcanology of La Palma and El Hierro, Western Canaries. *Estudios Geológicos*, **57(5-6)**, 175–273; <https://doi.org/10.3989/egeol.01575-6134>
28. Remigi, S., Frezzotti, M. L., and Ferrando, S. (2019). Generation of CO_2_-SO_2_ fluxes in the lithospheric mantle beneath El Hierro (Canary Islands) on metasomatic reactions of carbonate-rich silicate melts. In *XXV ECROFI Conference (European Current Research On Fluid Inclusions)* (pp. 102-102).
29. Gass, I. G. (1970). The evolution of volcanism in the junction area of the Red Sea, Gulf of Aden and Ethiopian rifts. *Philosophical Transactions for the Royal Society of London. Series A, Mathematical and Physical Sciences*, **267(1181)**, 369-381; <http://www.jstor.org/stable/73627>
30. Mohr, P. (1983). Perspectives on the Ethiopian volcanic province. *Bulletin volcanologique*, **46(1)**, 23-43; <https://doi.org/10.1007/BF02598243>
